# Biopolymer-Modified Carbon Paste Electrode for the Electrochemical Detection of Pb(II) in Water

**DOI:** 10.1155/2022/5348246

**Published:** 2022-01-31

**Authors:** Ousama Ifguis, Abdelaziz Moutcine, Charaf Laghlimi, Younes Ziat, Rachid Bouhdadi, Abdelilah Chtaini, Amine Moubarik, Mohamed Mbarki

**Affiliations:** ^1^Engineering Laboratory in Chemistry and Physics of Matter, Faculty of Science and Technics, Sultan Moulay Slimane University, Beni Mellal, Morocco; ^2^Electrochemistry and Molecular Inorganic Materials Team, Sultan Moulay Slimane University, Faculty of Sciences and Technology in Beni-Mellal, Beni Mellal, Morocco; ^3^Applied Chemistry Team, Faculty of Sciences and Techniques of Al Hoceima, Abdelmalek Essaadi University, Tetouan, Morocco; ^4^Engineering and Applied Physics Team (EAPT), Sultan Moulay Slimane University, Beni Mellal, Morocco; ^5^Laboratory of Chemical Processes and Applied Materials, Sultan Moulay Slimane University, Polydisciplinary Faculty, Beni Mellal, Morocco

## Abstract

During the present study, biopolymer lignin was extracted, in particular, from sugar beet pulp (molasses) from the Tadla region (224 km from Marrakech, Morocco). The lignin was characterized by infrared spectroscopy (FTIR) and thermogravimetric TG/DTA analysis and then used as a modifier to enhance the electroanalytical detection of heavy metal ion traces. The performance of the lignin/CPE sensor to detect lead (II) was studied by cyclic voltammetry (CV) and square-wave voltammetry in 0.3 mol L^−1^ NaCl. With optimized experimental parameters, the lignin/CPE sensor developed has a minimum detection limit of 2.252.10^−11^ M for Pb (II). The proposed working electrode has been successfully applied for the coanalysis of Pb (II) in tap water with good results.

## 1. Introduction

Faced with the industrial, urbanistic, and demographic development of the urban agglomeration, industrial effluents containing heavy metals at concentrations that exceed standards, even in trace amounts, are likely to create significant nuisance to individuals and their environment [1]. It is, therefore, essential to eliminate these toxic elements or to reduce their quantity below the admissible thresholds defined by the standards [2–4]. Different studies have been conducted on the detection of traces of some heavy metals, namely, indirect detection of dichromate in aqueous solution [5], determination of Sn (II) in real samples [6], and removal of Cu (II) from aqueous solutions in continuous and batch flow systems based on clinoptilolite nanoparticles modified with cysteine [7].

Several analytical methods can be used for the determination of heavy metals in aqueous solutions such as Pb (II), Hg (II), and Cu (II), such as atomic adsorption spectroscopy [4], the combination of cold vapor generation and inductively coupled plasma mass spectrometry (CV-ICP-MS) [8], and inductively coupled plasma optical spectrometry (ICP-OES) [9]. These techniques use complex and expensive instruments and are not suitable for in situ analysis or for sample pretreatment [10, 11]. On the other hand, electrochemistry is considered as an alternative and cost-effective method [12–14]. Electrochemical methods have been widely used for the determination of various heavy metals such as Hg (II) and other chemical compounds due to their outstanding advantages (simplicity, speed, low cost, efficiency, miniaturization capability, etc.) [15, 16].

In this context, different research works have been carried out, namely, the electrochemical determination of trace amounts of mercury in a water sample using an EDTA-CPE-modified electrode [17], preparation, characterization, and selective determination of ultratrace Pb(II) in water samples [18], and simultaneous determination of trace levels of Hg (II) and Pb (II) ions in various samples using a modified carbon paste electrode based on multiwalled carbon nanotubes and a new synthesized Schiff base [19]. Environmental pollution by Pb (II) is a very serious problem for fauna and flora as well as for human health [20]. Lignin is a three-dimensional amorphous polymer composed of methoxylated phenylpropane structures [21–24]. We found lignin binds to the cell walls, and its relative abundance is approximately between 20 and 30% of the total mass. Lignin is a thermoplastic polymer [12] used to make composites. Globally, about 50 million tons of lignin is produced annually as a residue from paper manufacturing processes [25]. Most of the lignin residue is burned to generate energy. However, on the basis of its interesting functionalities and properties, lignin offers several application possibilities, namely, combustion, as an additive in cement (cement setting retarder), and as an additive in asphalt (because of its antioxidant characteristics) [26, 27].

The main objective of this work is to modify a carbon paste electrode with molasses lignin under mild conditions to study the adsorption and sensing capacity of certain cation metals (Pb (II), Hg (II), and Cu (II)) simultaneously. Indeed, these heavy metals are known for their toxicity and have harmful effects on the nervous system, kidneys, and liver.

In this work, lignin was extracted from a biomass which is the molasses of sugar beet from the Béni Mellal region (191 km north of Marrakech, Morocco. The extracted lignin is used to modify a carbon paste electrode (CPE) to detect heavy metals in drinking water. The lignin-modified electrode/CPE sensor is capable of analyzing heavy metals in drinking water in order to make decisions on the sensitivity and stability of this sensor. Experiments conducted on this sensor show good results.

## 2. Materials and Methods

### 2.1. Preparation of Lignin

The lignin used as a basis for this study is “Beet pulp: Molasses.” Beet pulp is obtained after several steps of extraction of food sugar. The residue is used as a source of biomass for lignin extraction.

The beet pulp is presented in the form of small pieces of fibers of about 5 to 6 cm, after drying in the oven at 100°C for 48 hours, and then crushed using a mill brand “MONBROY, type MULTI-USAGE.” The obtained substrate is sieved, and only the 42.5 mm granulometry is kept.

An amount of 30 g of crushed material is placed in a crystallizer containing 300 ml of distilled water under stirring at 250 rpm at 80°C. After 1 h 30 min of treatment, the material is filtered and washed with distilled water to remove as many impurities as possible. The residue is rich in lignin and cellulose; however, the filtrate contains hemecellulose.

The second step is to isolate lignin and cellulose separately. To do this, 600 ml of NaOH (15%) is added to the purified substrate, under stirring at 250 rpm at 70°C for 1 h 30 min, to solubilize the lignin. To obtain the cellulose, the solution is filtered and washed. The obtained filtrate contains the soluble lignin.

In order to precipitate the lignin, the filtrate is neutralized with a solution of H_2_SO_3_ (5N) under stirring at 250 rpm. The whole process is controlled by using a pH meter, and this neutralization makes the medium more acidic (pH = 2).

Finally, the precipitated lignin is filtered to remove the liquid part, followed by washing with distilled water to remove traces of sulfuric acid. At last, the lignin is dried in an oven at 50°C for one day.

The modified electrodes were prepared by mixing carbon graphite powder and lignin (to obtain a ratio of 10/90 or 10% by weight of lignin). The kerosene oil supplied by Sigma-Aldrich is used as a binder. The resulting paste is introduced into a cylindrical cavity with a geometric surface area of 0.1256 cm^2^. The electrochemical behavior of the electrodes developed is studied in the potential range between −1.5 V and 1.5 V at 50 mV/s in a 1 M NaCl solution.

**Figure 1:**
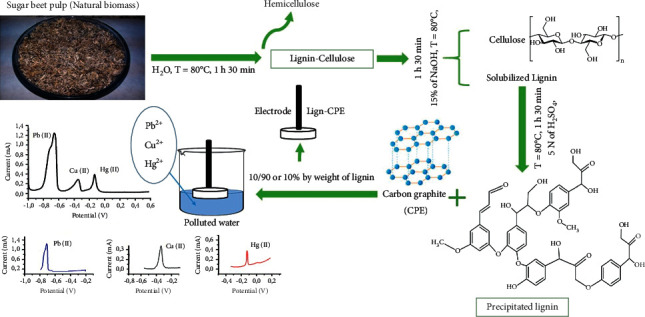
Preparation of the lignin-CPE electrode.

Figure 1 details the different steps of preparation of the lignin-CPE electrode.

### 2.2. Chemicals and Reagents

The carbon graphite powder and kerosene oil were supplied by Sigma-Aldrich, and the lead (II) chloride, mercury (II), and copper (II) were supplied by Riedel-de Haen. The extrapure sodium chlorine was obtained from Sharlau chemie (Spain). All solutions were prepared with distilled water.

### 2.3. Lignin Characterization

#### 2.3.1. TG/DTA Thermal Analysis

Thermogravimetric analysis is a thermal analysis technique that measures the quantity and rate of mass change of a sample as a function of temperature and/or time. The solid sample (Molasses) ground to 42.5 mm is introduced into the SETARAM Setsys TG 12 thermobalance after purging under air. The sample undergoes a temperature rise program of 5°C per minute from 25°C to 790°C.

#### 2.3.2. FTIR Spectroscopy

The analysis is carried out using a NICOLET 6700FT-IR spectrophotometer and a system of data acquisition developed by the brand.

The sample is placed directly on the ATR accessory single crystal which is crossed by the infrared beam which is reflected on the sample surface at an angle of 45° and then passes again through the crystal towards the detector. The spectra are recorded by performing 16 acquisitions between 400 and 4000 cm^−1^.

### 2.4. Device

The electrochemical measurements were performed in an electrochemical cell with three electrodes, a working electrode (WE) composed of lignin-modified carbon paste electrode (CPE-Lig), a saturated calomel reference electrode (RE), and an auxiliary electrode composed of platinum wire (AUX-E). A potentiostat (model PGSTAT 100, Eco chemie BV, Utrecht, the Netherlands), driven by a general electrochemical system, was used for data processing by software Volta lab master 4. A pH meter (HANNA HI 2210) was used for all pH measurements.

### 2.5. Working Electrode

The modified CPE-Lig electrode was prepared manually by mixing molasses lignin and graphite powder with kerosene oil, ethanol was added to the mixture as an inert and volatile solvent, and the resulting mixture was well homogenized by thoroughly mixing the two materials, and then, this mixture was returned to air for solvent evaporation. The resulting composite material (paste) was housed in a cylindrical PTFE tube, with the geometric surface area of the working electrode being 0.1256 cm^2^. A carbon bar ensured the electrical contact.

### 2.6. Measures and Procedure

The procedure followed for the electrochemical detection of the concentration of Lead (II) in aqueous samples consists in immersing the working electrode in a cell containing 3 *μ*M of analytical solution of Pb (II) of a selected pH and concentration.

## 3. Results and Discussion

### 3.1. Lignin Characterization

**Figure 2:**
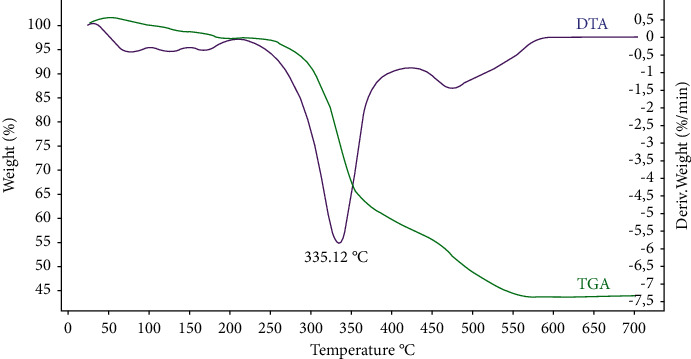
TG/DTA curve analysis of molasses lignin after separation at 5°C/min in air.

To study the thermal behavior of lignin, the Figure 2 shows the TG/DTG curve of lignin. TG/DTG curves show the presence of three different states of thermal degradation ranging from 25 to 700°C, providing valuable information on the thermal behavior of lignin at each step. Note that the first state shows that when the temperature is below ∼120°C, most of the weight loss in this state was due to the evaporation of free water from the lignin. In the second step, for molasses beet, partial pyrolysis of the lignin takes place at ∼200 to ∼400°C for molasses beet with a mass loss of about 40%. Lignin degradation end state isolated from molasses ranges from 440 to 570°C, during which a weight loss of about 10.3% is obtained. Liberation of CO_2_ starts at about 240°C and rises with a temperature up to 350°C; additionally, CO is liberated over a wide temperature range from 450 to 700°C, with a maximum at 550°C. Over a high temperature range from 450 to 700°C, some lignin derivatives such as phenolic, alcohols, and aldehydic acids, as well as the formation of gaseous products (CO, CO_2_, and CH_4_), are released.

**Figure 3:**
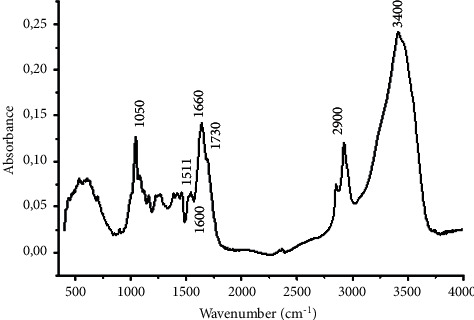
IR spectrum of lignin obtained.

The identification of the biopolymer lignin form was carried out by FTIR spectroscopy (Figure 3). Infrared spectroscopy of lignin is an analysis that reveals the presence of functional groups in lignin. The absorption band at 3400 cm^−1^ is attributed to hydroxyl groups. The region between 3000–2800 cm^−1^ corresponds to the C-H elongations of methyl and methylene groups, with the band at 2900 cm^−1^ corresponding to the C-H vibration of methoxy groups. The elongation vibration of the carbonyl group C = O is between 1740 and 1700 cm^−1^ for nonconjugated carbonyls and carboxylic acids and between 1650 and 1675 cm^−1^ for carbonyls located on conjugated structures. Vibration bands of the aromatic skeleton are observed at 1600, 1511, and 1420 cm^−1^. Finally, the region between 1300–1000 cm^−1^ corresponds to the vibrations of the various C-O and C-H bonds [28–30].

### 3.2. Potential Range of Application of Carbon Paste Electrodes

**Figure 4:**
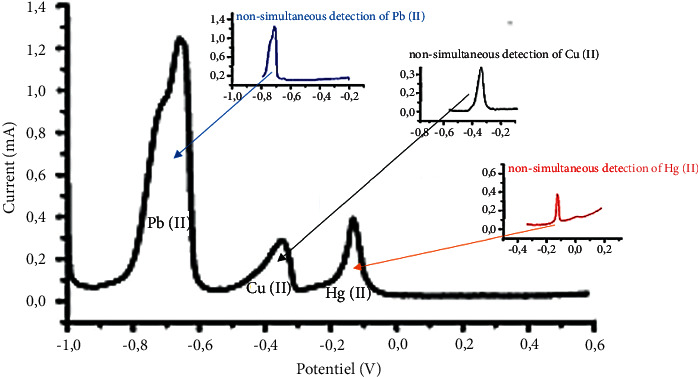
Nonsimultaneous and simultaneous detection of the three ions Pb (II), Cu (II), and Hg (II).

Figure 4 shows that the application potential range of the molasses lignin-modified carbon paste electrode is wide. This electrode is capable of sensing Pb (II), Hg (II), and Cu (II) ions in water with high sensitivity.

Thus, we notice that the peaks of the Pb (II), Hg (II), and Cu (II) ions are well separated, so the electrode can detect these metal ions simultaneously.

### 3.3. Electrochemical Behavior of Pb(II) Ions at the Unmodified CPE Electrode and the Modified CPE-Lignin Electrode

**Figure 5:**
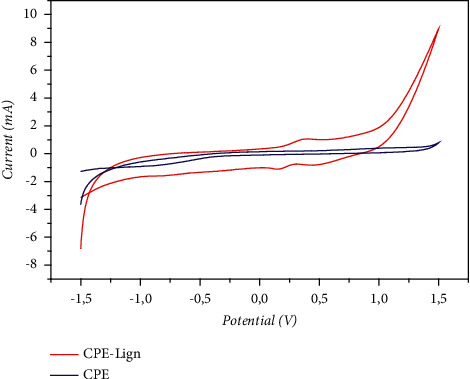
Cyclic voltammograms obtained at CPE and lignin-CPE in 0.1 M NaCl solution containing a scan rate of 0.1 Vs^−1^ with pH 2.5.

At the beginning, we recorded the cyclic voltammograms (CV), relating to the electrodes, CPE (the mother electrode before modification), and the modified electrode (Lign-CPE). By comparing the voltammograms of the two electrodes (Figure 5), we can see that the pattern of the cyclic voltammograms is different in the two electrodes. This corresponds to a change in the morphology of the surface and confirms the modification of the surface by lignin.

The recorded voltammogram for the elaborated electrode (lignin-CPE) shows two redox peaks. The first one in the cathodic scanning direction at about 0.2 V and the second one in the anodic direction at 0.3 V. These peaks are attributed to the electrochemical response of the lignin film, which proves that the lignin attached to the electrode surface has redox properties.

**Figure 6:**
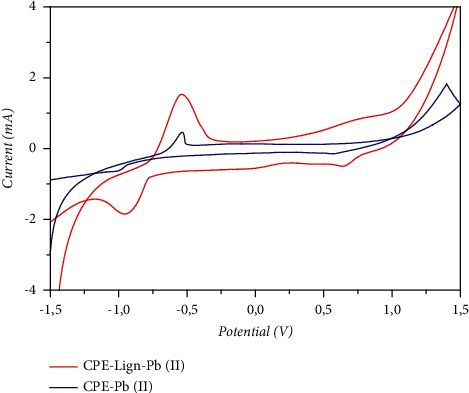
Cyclic voltammograms of modified lignin-CPE and unmodified CPE presence of 3 *μ*M of ions metals Pb (II) in 0.1 M NaCl as electrolyte, pH 2.5, and scan rate 100 mV/s.

Following impregnation in a solution containing lead ions, the electrode (Lign-CPE) is characterized in an electrolytic medium by recording the cyclic voltammogram (Figure 6). The cyclic voltammogram shows two peaks, the first in the anodic scanning direction −0.6 V and the second in the cathodic direction appears at −0.9 V, which correspond, respectively, to the oxidation of lead, adsorbed during the stay of the electrode in the lead solution, and to the reduction of lead ions. The observations made were able to reveal the reactions that occur on the surface of the modified electrode. During the preconcentration step, the ionic lead complexes on the surface of the modified electrode. At −0.9 V (cathodic scan direction), the already complexed Pb (II) ions reduce to metallic lead. In the anodic scan direction, the metallic lead oxidizes which explains the appearance of a peak at −0.53 V.

The proposed mechanism is represented as follows [31]:

  Organic matrix of the electrode Pb^2+^ + 2e^−^ ⟶ organic matrix of the electrode Pb
  Organic matrix of the electrode Pb ⟶ organic matrix of the electrode Pb^2+^ + 2e^−^

### 3.4. Analytical Parameter Optimization

In order to obtain the best analytical parameters and to improve the performance of the electrode (lignin-CPE) for sensing Pb(II) in aqueous solution, certain conditions were studied using square waves such as the supporting electrolytes, the preconcentration time, and the effect of pH and Pb(II) concentration.

#### 3.4.1. Supporting Electrolytes and pH Value

**Figure 7:**
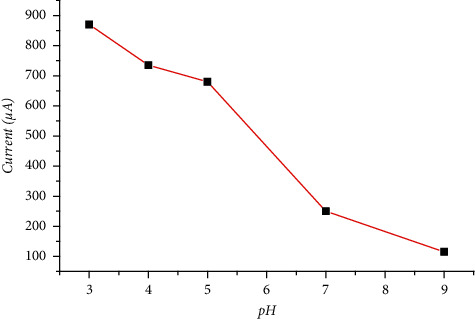
Influence of pH on the peak intensity response of Pb (II) ions at the lignin-modified CPE, at solution 0.1 MNaCl including 3 *μ*M of Pb (II) ion.

A study of the effect of pH on the voltammetric response of the lignin-modified carbon paste electrode was performed in the pH 3–9 range in a preconcentration solution containing 3 *μ*M lead. The maximum anodic current intensities were observed at pH = 3 (Figure 7). At basic pH, current densities decrease due to lignin denaturation.

The voltammograms show that if the pH of the solution decreases, the intensity of the anodic current peak increases, which proves that there is a participation of protons in the reaction at the electrode. It can, thus, be said that the intensity of the current peak depends on the pH of the solution.

The decrease in the intensity of the anodic peak in an electrolyte medium of pH 10 (basic medium) can be explained by the formation of lead hydroxide (Pb (OH)_2_), which leads to a decrease in Pb (II) ions on the surface of the modified electrode. On the other hand, the increase in peak current at pH = 2 (acidic environment) can be explained by the presence of the ionic form of Pb(II) which is dominant compared to lead hydroxide (Pb (OH)_2_) [31].

#### 3.4.2. Preconcentration Time Effect

**Figure 8:**
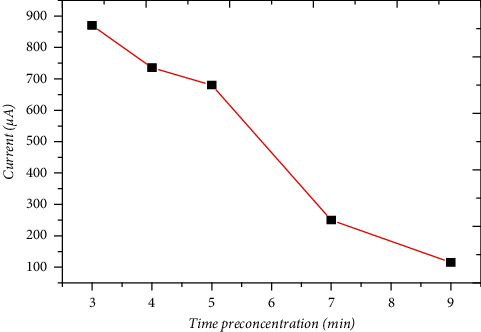
Influence of preconcentration time on the square-wave voltammetry response of Pb (II) at the lignin-modified CPE, at solution 0.1 M NaCl including 3 *μ*M of Pb (II) ion.

The influence of the preconcentration time on the current intensity of the lead anode peak was studied (Figure 8). This intensity decreases with the preconcentration time in particular, between 4 and 9 minutes. This indicates that there is a binding saturation of Pb (II) at the modified electrode and the phenomenon of salting-out [31]; for further manipulations, the preconcentration time adopted is 3 minutes.

### 3.5. Electrochemical Determination of Pb (II) by Lignin-Modified CPE

**Figure 9:**
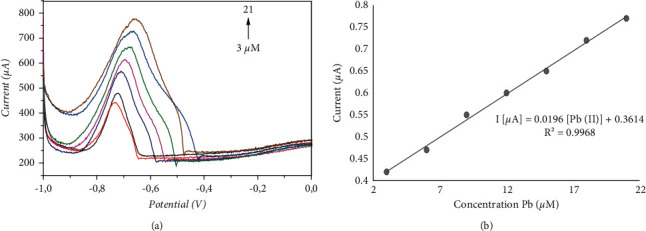
(a) Square-wave voltammograms recorded at different concentrations of Pb (II) in the range of 3 *μ*M to 21 *μ*M, in 0.1 M NaCl under optimal parameters (pH 9.0) using lignin-CPE. (b) Calibration curves of the lignin-CPE-modified electrode towards Pb (II) at different concentrations.

Once the optimal conditions of the parameters were determined, Pb (II) was detected on lignin-modified EPC using the square wave.  Figure 9(a) shows the curves (SW) for different concentrations of Pb (II) in the range of 3 *μ*M to 21 *μ*M, while the peak current of Pb (II) increases linearly with increasing concentration.

A linear equation was obtained, I [*μ*A] = 0.0196[Pb (II)] + 0.3614, *R*^2^ = 0.9968, Figure 9(b).

We have calculated the detection limit and the quantitation limit for the determination of Pb(II), respectively, using the following relationships (calculated by 3 *σ*/s and by 10 *σ*/s).

The detection limit is 2.252.10^−11^ M; the quantitation limit is 7.508.10^−11^ M.

According to previous studies on Pb (II) detection, our proposed lignin/CPE sensor has lower detection limits and good linearity as you can see in Table 1.

Therefore, the method meets the expectations of the reference measurement value and the detection limit of Pb (II) in the real sample. In addition, our method is advantageous because it requires fewer instruments, less operating time and costs, lower reagent consumption, and lower energy consumption than current spectrometric techniques.

Chronoamperometry can be used to determine the catalytic rate constant between the modified lignin-Pb electrode and Pb (II). From the chronoamperograms, the rate constant of the electrocatalytic reaction between the modified electrode and Pb (II) can be calculated according to the reduced form of the Galuse equation. The validation of the results for the electrodes can be studied by the statistical tests *t* and *g* [39].

### 3.6. Real Sample

In order to assess the electroanalytical of lignin/CPE-modified CPE in real sample, the determination of Pb (II) in tap water has been performed under optimal conditions, Table 2, as a selective and sensitive working electrode for the detection of Pb (II) without any sample pretreatment. The stripping peaks currents of Pb (II) were measured by SWAS. These results clearly show that the analytical performance of this sensor to Pb (II) can be quantified successfully with reliably and high precision [6, 11, 40].

## 4. Conclusions

The major objective of this work is to develop a new electrochemical sensor for the detection of Pb (II), Hg (II), and Cu (II) ions in tap water samples. The proposed modified electrode can be used to identify Hg (II) ions by square-wave voltammetry in tap water with a low DL 2.252.10^−11^ M after 9 min of preconcentration and good reproducibility. Such a sensor has a great power of use in order to improve portable analyzers for the sensitive detection of metal ions in a real sample. By such a new method, it is possible to promote selectivity and sensitivity. In addition, it is simple, fast, cost effective, and suitable for on-site analysis.

## Figures and Tables

**Table 1:** Comparison of the performance of the sensor developed with various electrochemical sensors previously reported.

Modified	Method	Pb(II)		Reference
		Linear range (*μ*M)	LOD (nM)	
SPGEs	SWASV	5–80	1.5	[32]
SH-SAAMS	ASV	0.2–1.6	3	[33]
SBA-15-MCPE	DPASV	2–10	400	[34]
Hg-MWCNT	ASV	—	—	[35]
SWCNT-PhSH/Au	ASV	5–90	3	[36]
AuNP-GCE	SWASV	0.2–1	10	[37]
MMT-Ca/CPE	SWASV	3.5–15	105	[38]
Lignin/CPE	SWASV	3–21	0.0225	This work

**Table 2:** Results for Pb (II) determination in tap water obtained under the optimum conditions.

Sample	Analyte	Added (*μ*M)	Found (*μ*M)	Recovery (%)
Tap water	Pb (II)	3	2,88	96

## Data Availability

All the results of this study are included in the document attached.
